# Trends of Hospitalization for Acute Alcohol Intoxication in Slovenian Children and Adolescents with and without Dual Disorder. Implications for a Correct Intervention

**DOI:** 10.3390/jcm9072122

**Published:** 2020-07-06

**Authors:** Mercedes Lovrecic, Barbara Lovrecic, Mateja Rok Simon, Ales Korosec, Filippo Della Rocca, Angelo G. I. Maremmani, Icro Maremmani

**Affiliations:** 1 National Institute of Public Health, 1000 Ljubljana, Slovenia; mercedes.lovrecic@nijz.si (M.L.); barbara.lovrecic@nijz.si (B.L.); Mateja.Rok.Simon@nijz.si (M.R.S.); Ales.Korosec@nijz.si (A.K.); 2 School of Psychiatry, University of Pisa, 56100 Pisa, Italy; filippo.dellarocca@yahoo.it; 3 Department of Psychiatry, North-Western Tuscany Local Health Unit, Tuscany NHS, Versilia Zone, 55049 Viareggio, Italy; angelo.maremmani@uslnordovest.toscana; 4 Association for the Application of Neuroscientific Knowledge to Social Aims (AU-CNS), 55045 Pietrasanta, Italy; 5 PISA-School of Experimental and Clinical Psychiatry, 56100 Pisa, Italy; 6 Vincent P. Dole Dual Disorder Unit, 2nd Psychiatric Unit, Santa Chiara University Hospital, University of Pisa, 56100 Pisa, Italy; 7 G. De Lisio Institute of Behavioral Sciences, 56100 Pisa, Italy

**Keywords:** trends of hospitalization, acute alcohol intoxication, psychiatric manifestations, public health, hospitalizations due to the toxic effects of alcohol, hospitalizations due to behavioral/mental issues, dual disorder

## Abstract

Background: Binge drinking and other forms of ethanol abuse are, when present, a serious problem in preteens and adolescents worldwide. Aim: The present study has analyzed the trend in alcohol-related intoxications requiring the hospitalization of children, adolescents and young adults aged less than 21 years in Slovenia in the 1999–2018 period. Methods: We performed a retrospective study on patients discharged after hospitalizations due to mental and behavioral disorders due to acute alcohol intoxication (MBDAAI) or hospitalizations due to the toxic effects of alcohol (TEA We considered three groups: children (aged 10–14), adolescents (aged 15–19) and young adults (20–21 years old). Hospitalization rates and time trends were analyzed using joinpoint regression to obtain the annually calculated age- and sex-specific rates and the annual percentage of change (APC). Results: Considering a total of 2912 MBDAAI-hospitalizations, 15–19-year-old subjects showed a significantly higher hospitalization rate compared to the immediately younger and older age groups and a significant increase in hospitalization rates in the period 1999–2011, followed by a significant decrease. Considering 1143 TEA-hospitalizations, we observed a continuous decrease in the hospitalization rates for children and young adults and, conversely, a continuous even if less than significant increase for adolescents aged 15–19. Conclusions: Alcohol consumption in Slovenian children and adolescents is a highly important health concern. Special attention to public health problem of severe alcohol abuse requiring hospitalization in children and adolescents is needed, especially with possible crisis of SARS-CoV-2/Covid-19 situation.

## 1. Introduction

Despite its abuse potential, ethanol is broadly tolerated in a social context and is therefore to be found everywhere in social settings. Alcohol use creates risks of around 230 different identified health outcomes taking the form of disease or injury, including infectious and non-communicable diseases (according to the International Statistical Classification of Diseases and Related Health Problems—10th Revision—ICD-10) [1,2]. Alcohol abuse is responsible for at least 60 major types of systemic diseases [3], while alcohol consumption increases the overall risk of developing cancer [4]. According to WHO, alcohol abuse gives rise to serious public health concerns, especially in the European region as defined by WHO [5], where alcohol intake (per capita alcohol consumption) is the highest in the world (so providing an indirect indicator of the severity of the burden due to alcohol-related diseases). The available data definitely point to higher levels of the disease burden attributable to alcohol use compared with other regions outside Europe [6]. Alcohol misuse among young people is widely recognized as a global health priority [5]. Ethanol use and abuse among young people (under 21 years of age) is a pervasive problem worldwide. Alcoholic beverages contain ethanol, a psychoactive drug [7] with relaxant and euphoric effects [8]; prolonged exposure leads to addiction.

Alcohol intoxication often develops as a sequence of urgent situations that call for a specific medical procedure and spring from a clinically harmful condition that is due to exposure to alcohol, when alcohol and its metabolites accumulate in the blood stream faster than they can be metabolized by the liver. In children and adolescents, alcohol intoxication usually proves to be a symptom of a broader problem requiring further investigations to identify potential problems that need special attention. In adolescents, alcohol use is likely to be episodic and to involve larger volumes (often taking the form of binge drinking) than those of adults. This creates a dangerous situation, with subjects running a high risk of alcohol overdose or alcohol poisoning. In such cases, suppression of the gag reflex and respiratory drive, typically aggravated by hypoglycemia, can be fatal [9]. Adolescence is a time of vulnerability and adjustment [10], accompanied by various risky behaviors, including experimentation with the initial stage of alcohol consumption, when impulse control is still relatively immature [11]. Risk of exposure to alcohol in adolescence is positively associated with the parental provision of alcohol, favorable attitudes towards alcohol use and drinking/misuse. On the other hand, it is negatively associated with parental monitoring, support and involvement in the framework of a high-quality parent–child relationship. The former, positively associated features have been identified as longitudinal predictors both of alcohol initiation and high levels of later alcohol use/misuse [12]. Brain development continues during adolescence and is particularly vulnerable to the effects of alcohol. Adolescent drinking lowers orbitofrontal cortex activity and raises amygdala activity, leading to less executive control, greater emotional impulsivity and alterations in decision-making. What is more, it raises the likelihood of engaging in risky behaviors and developing mental health problems later in life [13,14,15]. Early initiation into drinking, with the consequent risk of contracting alcohol use disorder, could be related to manifestations of psychopathology [16,17]. Exposure to alcohol in adolescence may lead to the retention of adolescent phenotypes (adolescent-typical responses to alcohol that last into adulthood). After alcohol intoxication, cognitive, behavioral and affective consequences have been reported. These include the impaired performance of executive functions, memory impairment, reduced cognitive flexibility, a preference for disinhibition and for running greater risks and conspicuously elevated social (in some cases general) anxiety. It should be added that some of these traits are specific to alcohol exposure during adolescence; they are not, in fact, evidently linked to the exposure to alcohol that occurs during adulthood [8,10,11].

Slovenia is a country with a long tradition of high per capita alcohol consumption registered among subjects over 15 years of age, currently remaining one of the countries with the highest per capita consumption [18]. Alcohol-related mortality in Slovenia is high, so much so that the age-standardized death rate (15+) for liver cirrhosis was 31.2 per 100,000 population in 2016 in Slovenia [19]. For decades now, Slovenia has continued to rank as a country with a higher per capita alcohol consumption (15+ years), a higher alcohol-related mortality (liver cirrhosis) and a rate of exposure to alcohol among those under 18 higher than the European averages (whether those for the EU or the WHO-defined “European region”) [20].

The 2015 European School Survey Project on Alcohol and Other Drugs (ESPAD) revealed that 15–16-year-old students in Slovenia reported above the ESPAD average for alcohol use in the last 30 days and heavy episodic drinking during the same period of time. Reported alcohol use in Slovenia (prevalence of lifetime use, 30-day use and intoxication) was (for boys, girls and total) above ESPAD average, same frequency of alcohol intake in the last 30 days by gender (mean number of occasions among users). Average alcohol intake on the last drinking day (centiliters of ethanol) and prevalence of five or more drinks (one drink contains approximately 2 centiliters of ethanol) at least once in the last 30 days by gender among users were above average for boys. Slovenia was among countries with stable or increasing lifetime prevalence in alcohol use during 2002 and 2010 period. Early onset of alcohol use as prevalence of students experiencing alcohol use and intoxication (for boys) at the age of 13 or younger (percentage) was above ESPAD average [21]. According to the Health Behavior in School-aged Children (HBSC) study conducted in 2014, 15-year-old Slovenian boys and girls were above European average in weekly drinking of alcohol, drunkenness initiation and drunkenness. This means they reported being drunk on two or more occasions, drinking alcohol at least once a week and reported first drunkenness at 13 years or younger. A survey in 2001 also showed that more 15-year-old Slovenians drank alcoholic beverage weekly in comparison to the European average. Other two observed age groups from the surveys (11 and 13 years old) were always close to the European average. Especially concerning is the information that in 2014, 4% of 11-year-old girls and 6% of boys the same age in Slovenia drank alcohol weekly (vs. 2% and 4% for European average) [22,23].

Data from literature revealed that hospital admissions due to alcohol intoxication in children generally increased by age, with boys being more likely than girls to be admitted in hospital and admissions increase among children and adolescents [24,25,26,27,28,29,30].

Among systemic measures directed towards alcohol related harm reduction in Slovenia, the most important is the Restrictions on the Use of Alcohol Act adopted in 2003 [31]; however, access to alcohol for children and adolescents (in spite of ban on the sale and dispensing of alcohol to minors) seems to be easy. On the other hand, Slovenia does not have sufficient services for the provision of outpatient care for children and adolescents with mental health problems [32]. In addition, only in 2018, ten years after adopting the Mental Health Act, the Slovenian Parliament [33] adopted the Resolution on the National Mental Health Program 2018−2028 (planned in Mental Health Act from 2008) with Action plan [33]. Both strategic documents adopted in 2018 include reform of mental health care and in a special way address alcohol related mental disorders and mental care for children and adolescents; however, both are not yet implemented and are still nowadays topics of discussion.

Collaboration between all services for the provision of care for children and adolescents with mental health problems, especially psychiatric and drug addiction services, should be optimized in such a way as to limit hospitalizations. Worsening trends over the years, as well as differences in hospitalizations regarding acute alcohol intoxication in patients with and without a dual disorder, can stand as indirect proof of difficult services integration. Studying this issue in adolescents can provide further indications of the efficacy of efforts to prevent psychiatric complications and drug abuse in Slovenia.

The aim of the present study was to examine the hospitalizations due to exposure to alcohol and analyze the trend in alcohol-related intoxications requiring the hospitalization of children, adolescents and young adults aged under 21 in Slovenia in the 1999–2018 period, considering the two main types of hospitalization according to the ICD-10 description of diagnosis.

We have considered hospitalizations due to behavioral/mental issues (MBDAAI) using ICD-10 F10.0 diagnostic criteria and those due to the toxic effect of alcohol (TEA) using the ICD-10 T51 ones.

## 2. Methods

### 2.1. Data Sources and Procedures

We have performed a retrospective epidemiological study using the data from the National Hospital Health Care Statistics Database (NHHCSD) (SI: Spremljanje bolnišničnih obravnav—SBO). The data from NHHCSD comprise episodes from all acute hospital care provided at national level. The NHHCSD is managed by the National Institute of Public Health and encompasses data on hospital discharges including discharge diagnoses, procedures and the demographic characteristics of patients. NHHCSD is implemented at the national level, being mandatory (laid down by law), so that all hospitals in the country are included, and the system has been functioning uninterruptedly for decades.

All the hospitalization cases recorded in the 1999–2018 period, where patients were examined at the time of admission, were aged between 10 and 21, inclusive. They had a main discharge diagnosis classified as F10.0 (Mental and behavioral disorders due to acute alcohol intoxication) or T51 (Toxic effects of alcohol), in accordance with the criteria of the International Statistical Classification of Diseases and Related Health Problems—10th Revision (ICD-10).

The data were acquired in an aggregated and anonymized form (number of hospitalizations specified by year, diagnosis, sex and age group), so that no permission from the Republic of Slovenia Ethics Committee was needed for this study.

### 2.2. Statistical Analysis

For analytical purposes, patients were divided according to sex and into distinct age groups: 10–14, 15–19 and 20–21 years. We chose these three age groups because of different alcohol-use patterns and of Slovenian legislation that takes three different forms on the basis of the age group in question. In addition, a different type of attention is paid by the Slovenian National Health System, which provides a different type of health collaboration for each of the three groups. The annual age or age- and sex-specific crude hospitalization rate for each main diagnosis (T51 or F10.0) was calculated by dividing the number of hospitalizations recorded in each year by the corresponding number of inhabitants as of 1 July each year (data acquired from the Statistical Office of the Republic of Slovenia) and expressed per 100,000 inhabitants. For hospitalization rates, the exact 95% Poisson confidence intervals were calculated, and the 1999–2018 average crude hospitalization rate was compared with the rate in each year, separately for each sex and diagnosis.

By using joinpoint log-linear regression, we identified the years in which changes in trends occurred in the rates for hospital admissions for alcohol-related intoxications and estimated the annual percentage change (APC) in each of the periods. For each segment, the figure for the annual percentage change revealed whether it was significantly different from zero. All the combinations of sex- and age-group models with zero to three joinpoints were tested, and the final model with the lowest number of statistically different trend segments was pre-selected and critically evaluated. The software in use was the Joinpoint Regression Program, V 4.7.0.0 (Statistical Research and Applications Branch, National Cancer Institute).

## 3. Results

### 3.1. Mental and Behavioral Disorders Due to Acute Alcohol Intoxication (MBDAAI; F10.0)

**Figure 1:**
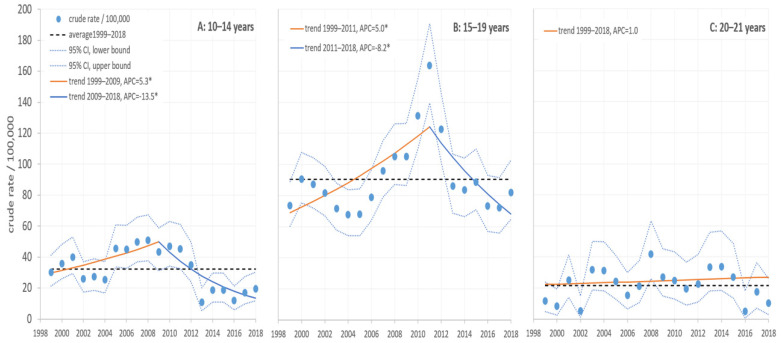
Hospitalization rates (/100,0*00) for patients with mental and behavioral disorders due to acute alcohol intoxication (MBDAAI) in Slovenia during the 1999–2018 period.

We identified 2912 hospitalizations (1662 males and 1250 females) MBDAAI, which had been identified as the main diagnosis among patients aged 10–21 in the years 1999–2018 inclusive (Figure 1).

In the 10–14-year age group, during the period 1999–2009, a significant increase in the hospitalization rate for MBDAAI of 5.27% per year (*p* ≤ 0.05) was observed. Conversely, in the years 2009–2018, that rate decreased by as much as 13.55% per year (*p* ≤ 0.05).

In the 15–19 age group, the hospitalization rate rose significantly, by 5.02% per year (*p* ≤ 0.05) in the period 1999–2011; it then fell by 8.23% per year (*p* < 0.05) in the years 2011–2018.

In the 20–21 age group, there was a continuous increase in the hospitalization rate of 1.02% per year throughout the study period for those patients, but the trend failed to reach the threshold of significance (*p* > 0.05).

Adolescents (aged 15–19) had a significantly higher hospitalization rate compared to the immediately younger age group (10–14 years) (OR = 2.78; 95%, CI = 1.88–4.12; *p* < 0.001) and again compared to the immediately older (20–21 years) age-group (OR = 4.10; 95% CI = 2.20–7.63; *p* < 0.001).

No significant gender-related differences were found in or between groups.

### 3.2. Toxic Effects of Alcohol (TEA; T51)

**Figure 2:**
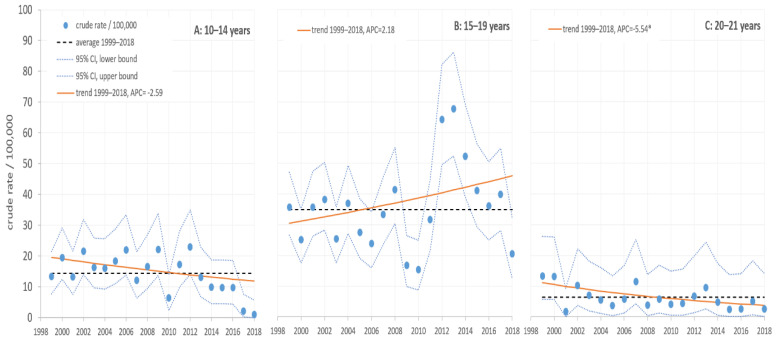
Hospitalization rates (/100,0*00) for toxic effect of alcohol (TEA) patients in Slovenia during the 1999–2018 period.

We also identified 1143 hospitalizations (638 males and 505 females) arising from TEA as the main diagnosis among patients aged 10–21 years in the period 1999–2018 (Figure 2).

In the group aged 10–14, during the period 1999–2018, a continuous decrease in the hospitalization rate for TEA by 2.59% per year (*p* > 0.05) was observed.

In the group aged 20–21, during the study period, a significant continuous decrease in the hospitalization rate by 5.54% per year (*p* ≤ 0.05) was noted.

By contrast with the previous two groups, for those aged 15–19, the hospitalization rate continuously increased by 2.18% per year (*p* > 0.05).

Adolescents (aged 15–19) had a significantly higher hospitalization rate both than the immediately younger (10–14 years) (OR = 2.34; 95% CI = 1.29–4.24; *p* = 0.005) and immediately older (20–21 years) age groups (OR = 5.74; 95%CI = 1.78–18.58; *p* = 0.004).

In this case also, no significant differences were found between males and females in or between our study groups.

## 4. Discussion

Generally speaking, all our study patients with MBDAAI had higher hospitalization rates than those with TEA, especially regarding the 15–19 age group patients, who showed a shift in hospitalization rate trends in 2011 and had significantly higher rates compared to the immediately younger and older age groups. For the 10–14 age group patients, the trend was similar.

At the same time, TEA-hospitalizations showed a continuous falling trend during the entire study period in the 10–14 and the 20–21 age groups, but for subjects aged 15–19, the results turned out differently. In particular, adolescents (aged 15–19) had a significantly higher TEA-hospitalization rate compared to the immediately younger and older age groups and showed a continuous increase in the TEA-hospitalization rate observed throughout the study period. In Slovenia, there is a high prevalence (among countries with an incidence of over 30%) in heavy episodic drinking among young people aged 15–19 [12]. ESPAD international surveys 2015 revealed that 15–16-year-old students in Slovenia reported above the ESPAD average in current and past alcohol use, initiation and intoxication at the age of 13 or younger, while Slovenia was among countries with stable or increasing lifetime prevalence in alcohol use during 2002 to 2010 period. The prevalence of lifetime alcohol use between 1995 and 2015 remained little changed in Slovenia; however, the use of any alcoholic beverage during the past 30 days has decreased in most countries [21]. HBSC 2014 revealed that Slovenian 15-year-old boys and girls were above European average in weekly drinking of alcohol, drunkenness initiation and drunkenness. Already in 2001, more 15-year-old—and in 2014 more 11-year-old—Slovenian children drank alcoholic beverages weekly in comparison to the European average [22,23].

On the other hand, only a small percentage of adolescents were admitted to hospitals to treat alcohol intoxication [21]. The data for MBDAAI- and TEA-hospitalizations could be defined as being only the tip of the iceberg of the problem of the exposure of children and adolescents to alcohol, and estimated numbers of unreported cases in Slovenia are higher.

Hospital admissions due to alcohol intoxication in children and adolescents increased by age; boys were more frequently hospitalized, and similar characteristics were reported by other studies [24,26,27,28,29,30,34].

In Germany, it was reported that, between 2000 and 2011, there was an increase of over 170% in hospital treatment of adolescents’ alcohol intoxication [35]. A number of studies have shown an increase in the number of hospital treatments for alcohol intoxication in children, especially for adolescents [24,26,27,28,30].

Other authors reported different trends in numbers of hospitalizations due to alcohol intoxication in adolescents between 2000 and 2010, with an increase in the 2000–2007 period, then a decrease until 2010; in any case, altogether, hospitalizations were higher in 2010 than in 2000 [25].

Of particular concern were the data regarding the MBDAAI-hospitalization rate for patients aged 10–14, during the period 1999–2009. A significant increase in the hospitalization rate was observed. On the other hand, during the 1999–2018 period, a continuous decrease in the hospitalization rate for TEA was observed. For patients aged 20–21, considering the 1999–2018 observation period, a continuous increase in the MBDAAI-hospitalization rate was observed, while the TEA-hospitalization rate in the same period showed a significant decrease. In our study, gender differences were not statistically significant, in line with the results of previous studies [36,37]. However, gender differences have been observed regarding alcohol intoxication characteristics. In particular, intoxicated girls tended to be younger, had a lower blood alcohol concentration and were hospitalized for shorter times than boys [25].

Several studies regarding hospitalizations of adolescents with alcohol intoxication have been performed in Germany and the Netherlands.

Hospital-admitted adolescents were, in general, 12–18 years old, with an average age of 15.3 years. Intoxicated adolescents appear to be a representative sample of the Dutch population regarding all background variables (gender, educational level, family structure) indicating that more strict governmental alcohol control policies are required [37].

Again, regarding the Netherlands, the number of adolescents suffering from alcohol intoxication increased in 2008 compared to 2007, probably being related to the parental (lack of) involvement with and responsibility for commercial sales personnel [38].

Another naturalistic study confirmed that known risk factors for the development of Alcohol use disorders (AUD) also apply to adolescents hospitalized for alcohol intoxication. This finding facilitates targeted prevention efforts for adolescents who need more than a standard brief intervention for aftercare [39].

In a multicenter study, in the majority of adolescents, further development until their mid-twenties appeared to be unremarkable. However, their risk of developing severe AUD or other problematic outcomes was significantly greater [40]. The severe nature of adolescent intoxication has often been reported, indicating that more strict governmental alcohol control policies are required [37].

In the Americas, Europe and the Western Pacific Region, where alcohol use starts before the age of 15, the prevalence of alcohol use among 15-year-old students is reported as falling within the range of 50–70%, with only minor differences detected between boys and girls. As many as 26.5% of all adolescents aged 15–19 are exposed to alcohol (actually qualifying for being called “current drinkers”), so reaching a total of around 155 million adolescents worldwide. Prevalence rates of current drinking are highest among 15–19-year-olds in the WHO European Region (43.8%), followed by the Region of the Americas (38.2%) and the Western Pacific Region (37.9%). Worldwide and in all WHO regions, the prevalence of heavy alcohol drinking is lower among adolescents (aged 15–19) than the general population, but it peaks in the 20–24 years age bracket, when its frequency exceeds that in the total population. All the high prevalence rates for heavy alcohol drinking among young people aged 15–24 exceed the rates recorded in the total population (the only exception being the Eastern Mediterranean Region). Current drinkers in the 15–24 year age bracket often indulge in heavy drinking sessions, with a particularly high prevalence among males [41].

Every instance of alcohol intoxication in children, whether in the preadolescent or adolescent period, raises concerns, because early exposure to alcohol and binge drinking is associated with a wide spectrum of short-term and long-term negative consequences, including blackouts, hangovers and alcohol poisoning, injuries incurred because of alcohol and drug use disorders, other mental disorders, a risk factor for suicide attempts and other pathologies, carcinogenic risks, a greater risk of becoming a victim of assault or accidental death as well as risky sexual behaviors, academic problems, and delinquent behavior [42,43,44,45,46].

The data reported in our study indicate a serious risk for young people in Slovenia. The trend recorded for MBDAAI-hospitalizations is currently decreasing but still shows high levels, whereas the trend for TEA-hospitalizations is less alarming. However, all alcohol related hospitalizations in our case were 100% preventable and represent high risk for health and other short- and long-term consequences.

One possible explanation for the still high incidence of MBDAAI-hospitalizations and for the various trends observed over time in patients with Dual Disorder depends on differences in the attitudes of psychiatrists towards people with addiction and, similarly, on differences in ways of dealing with alcoholism. In one of our studies [47], we stressed that insufficient communication between general psychiatric and addiction services may lead to a phenomenon called “unreported double frequency”, which refers to the simultaneous attendance by patients of the two types of service, while therapists are left uninformed [48]. The reasons for that may be shame or manipulation (while patients may aim to acquire extra benefits and obtain the prescription of additional medications) resulting in less appropriate and less effective intervention. In addition, ethical considerations, legal complications, therapeutic relationships and insufficient collaboration between the health, social and psychiatric services may be involved [49,50,51,52,53,54,55].

In dual disorder alcoholics or in patients with psychiatric manifestations of alcoholism, the possibility that inadequate intervention may cause more frequent hospitalizations cannot be ruled out. On the other hand, the integrative approach to alcoholism may be responsible for the significant decline recorded in the number of MBDAAI-hospitalizations in children after 2009 and in adolescents after 2011, as well as for the continuous fall in the hospitalization rate for TEA in those aged 10–14 that was observed during the period 1999–2018. Improvements, such as decline in hospitalizations for acute alcohol intoxication, might be an important result of a law passed on the restriction of the use of alcohol, better preventive and educational measures in schools and media campaigns or the result of changing attitudes to alcohol drinking among children and youngsters. However, the economic crisis in 2009 in Slovenia should be considered, bearing in mind that during crises alcohol consumption usually decreases with decreased access, while binge drinking persists or increases. On the other hand, negative influences may have been exerted by an increase in the number of TEA-hospitalizations, and various other changes could have had an impact, e.g., the entry of Slovenia into the EU in 2004, the rapid expansion of various major grocery chains entering the market after 2005 in Slovenia with consequent greater choice and easy access to alcohol for minors (selling alcohol to minors), cheap beverage. Of particular interest is the increase of hospitalizations for TEA in 15–19-year-olds while in other two age groups there was a decrease (significant for group of 20–21-year-olds). Another explanation might be experimentation and binge drinking patterns and easy access to alcohol in this age group. In addition, Slovenia already faced two economic crises, first after the Slovenian independence in 1991 and second in 2009 in the wake of the outbreak of a global financial crisis. That negative outcome now runs the risk of repeating itself if the economic crisis following the present SARS-CoV-2/Covid-19 situation. In addition, it is common knowledge that during and after crises, the mental health of the population is impaired.

By improving the degree of collaboration between psychiatric services, addiction units and hospitals, a further reduction in the still high MBDAAI-hospitalization rate in Dual Disorder patients could be achieved. Our results should be a cause for reflection not only for centers of study on alcoholism or for those studying mental health. Pediatricians and health care providers for adolescents play an important role in assessing the risks that derive from severe alcohol use and in identifying substance use by young people [56,57]. According to the recommendations made by the American Academy of Pediatrics to pediatricians to conduct routine annual substance use screening of all adolescents [58], there are two quick and validated screening tools for determining substance use disorders in adolescents—the Alcohol Use Disorders Identification Test (AUDIT) developed by the World Health Organization and the CRAFFT (a mnemonic acronym consisting of the first letters of the six key words appearing in the six screening questions: Car, Relax, Alone, Forget, Friends, Trouble) [59]. Lastly, in the emergency rooms of hospitals, the initial treatment of acute intoxication due to drinking alcohol should be followed up at a later stage by brief motivational sessions dedicated to persuasive intervention [60,61]. Early and more frequent psychological intervention could, in fact, account for a decreasing trend in alcohol intoxications in young people. In addition, the prohibition of alcohol sales to young people should be more efficiently conducted and supervised. There is also a need to upgrade the law on the restriction of the use of alcohol in Slovenia adopted 17 years ago; systemic interventions to limit access to alcohol should be updated based on evidence. Access to psychiatric services for children and adolescents should be guaranteed, and the needs of this population should be meet.

The strong points of this study included its collection of national data (all the hospitals in Slovenia reported their available data), making use of decades of routinely implemented information systems and the mandatory reporting of data as required by law, treatment within Slovenia’s national health system (in a hospital environment) and full implementation of the diagnostic process, independently of any self-reporting. As there has not been any official change in our admission policy, we believe that our data show the actual trend of alcohol consumption in children and adolescents in our region.

This study’s limitations include insufficient data on the places and times when alcohol consumption occurred, types and quantities of alcohol drinks or other concomitant drug use. We were unable to trace data on the reasons and circumstances that had led to alcohol intoxication or data about another concomitant psychopathology. The numbers of children, adolescents and young adults intoxicated by alcohol are probably higher than those recorded here, as our database did not include cases with only minor signs of alcohol intoxication or situations when the victims of intoxication had not requested medical treatment (e.g., some patients were treated at home). It was not feasible to assess consequences or find details about further treatment(s) after hospital discharge.
